# The role of recombinant LH in women with hypo-response to controlled ovarian stimulation: a systematic review and meta-analysis

**DOI:** 10.1186/s12958-019-0460-4

**Published:** 2019-02-06

**Authors:** Alessandro Conforti, Sandro C. Esteves, Francesca Di Rella, Ida Strina, Pasquale De Rosa, Alessia Fiorenza, Fulvio Zullo, Giuseppe De Placido, Carlo Alviggi

**Affiliations:** 1 Department of Neuroscience, Reproductive Science and Odontostomatology, University of Naples Federico II, Naples, Italy; 2 ANDROFERT, Andrology and Human Reproduction Clinic, Campinas, Brazil; 3 Department of Senology, Medical Oncology, National Cancer Institute, IRCCS “Fondazione G.Pascale”, Naples, Italy; 4 Unit of Obstetrics and Gynaecology, Department of Experimental and Clinical Medicine, Magna Graecia University of Catanzaro, Catanzaro, Italy; 5 Istituto per l’Endocrinologia e l’Oncologia Sperimentale (IEOS) Consiglio Nazionale delle Ricerche, Naples, Italy

**Keywords:** LH, Recombinant LH, Hypo-responders, Assisted reproductive technologies, In vitro fertilization, Ovulation induction

## Abstract

**Objective:**

To study the role of recombinant human LH supplementation in women with hypo-response to ovarian stimulation.

**Methods:**

We performed a systematic review and meta-analysis of prospective clinical trials in which recombinant FSH monotherapy protocols were compared with LH-supplemented protocols in hypo-responders. A search was conducted of the Scopus, MEDLINE databases without time or language restrictions. Primary outcome was clinical pregnancy rate.

**Results:**

Significantly higher clinical pregnancy rates (odds ratio: 2.03, *P* = 0.003), implantation rates (odds ratio: 2.62, *P* = 0.004) and number of oocytes retrieved (weight mean differences: 1.98, *P* = 0.03) were observed in hypo-responders supplemented with recombinant LH versus hypo-responders who underwent FSH monotherapy. No differences in terms of mature oocytes or miscarriage rates were found between the two groups.

**Conclusion:**

In conclusion, our analysis confirms that women with a hypo-response to exogenous gonadotropins might benefit from LH supplementation. However, more trials are required before a definitive conclusion can be drawn.

**Electronic supplementary material:**

The online version of this article (10.1186/s12958-019-0460-4) contains supplementary material, which is available to authorized users.

## Introduction

An appropriate ovarian response to controlled ovarian stimulation (COS) is crucial for the success of assisted reproductive technologies (ART) [1]. The number of oocytes retrieved at the end of stimulation is the parameter most often used to assess ovarian response to exogenous gonadotropin and is strictly related to live birth rate [2]. Based on ovarian biomarkers and oocyte number, women are classically defined as poor, normal or hyper-responders [1, 3]. However, there is a specific subgroup of women termed “hypo-responders” who have an unexpectedly poor or suboptimal response to gonadotropin therapy despite adequate ovarian pre-stimulation parameters [4, 5].

Hypo-responders are hyposensitive to standard age, ovarian biomarkers, and BMI-matched doses of exogenous FSH [4–6]. This ovarian resistance to gonadotropin stimulation might clinically manifest as an “initial slow response” [7, 8] or be retrospectively diagnosed in women who require higher-than-expected doses of gonadotropins on the basis of age, BMI, and ovarian reserve [9]. The hypo-responder patient differs from the classic poor responder patient in at least two aspects. First, in hypo-responders, the number of oocytes is retrieved is higher than three, albeit at the expense of elevated gonadotropin consumption, whereas in classic poor responders, the number of oocytes retrieved is usually lower irrespective of the amount of gonadotropin administered. Second, hypo-responders have normal ovarian reserve tests (i.e., anti-Müllerian hormone and antral follicle count), which are usually deranged in women with the classic poor ovarian response.

The reasons for the hypo-responsiveness to gonadotropin stimulation are not entirely understood, but it has been suggested that genetic mutations or single nucleotide polymorphisms (SNPs) of gonadotropins and their receptors might influence ovarian sensitivity to exogenous gonadotropins [6, 10–12]. Hypo-responders undergoing ART treatments might face increased treatment costs, decreased cumulative live birth rates, and increased time to live birth, suggesting a negative impact on fertility. Yet, is still unclear how to most optimally manage this group of patients, although recent evidence suggests that hypo-responders might benefit from recombinant LH (rLH) supplementation during ovarian stimulation [13–15]. Physiologically, LH activity is crucial for proper folliculogenesis. Indeed, in the late follicular phase, granulosa cells are receptive to LH which can sustain follicular growth even when exogenous FSH administration is discontinued [16]. While the indiscriminate use of LH supplementation remains equivocal in normal responders, who are characterized by a normal function of the gonadal axis, the addition of LH supplementation in hypo-responders may be clinically beneficial as these patients can harbor polygenic traits affecting the functional properties of endogenous gonadotropins and/or their receptors [11]. To verify these preliminary observations, we aggregated the available published data of prospective clinical trials on the effect of rLH supplementation in hypo-responders using meta-analysis. Our aim was to summarize the evidence on the clinical utility of adding rLH to COS in hypo-responders undergoing ART.

## Methods

### Search strategy

We conducted a systematic search using Medline/PubMed and Scopus databases to identify all relevant studies. The search terms used, alone or combined, were “luteinizing hormone”, “recombinant LH”, “rLH”, “rhLH”, “ovulation induction”, “assisted reproductive technology”, “ART”, “in vitro fertilization”, “IVF”, “steady response”, “hyporesponse” (Additional file 1: Table S1). Hand searches of relevant review articles and reference lists were carried out. No time restriction and language restriction were applied and the end date for all searches was March 2018.

### Eligibility and data extraction

We included only prospective clinical trials in which recombinant human FSH (rFSH) alone protocols were compared with rFSH + rLH supplementation in women undergoing IVF/ICSI with a hypo-response to exogenous rFSH monotherapy. Hypo-response was defined according to authors’ criteria of included studies provided they met with our inclusion criteria. Specifically, hypo-responders were normogonadotropic women who required an elevated total dose of rFSH (> 2500 IU) to obtain an adequate number of oocytes retrieved or who had a plateau on follicular growth with no or only marginal increase in the estradiol level and in the follicular size during stimulation. Data extraction was performed independently by three authors (IS, PD and AF) using predefined data fields.

### Outcome measures

The primary outcome was clinical pregnancy rate (defined as the number of clinical pregnancies, i.e., presence of one or more gestational sacs with foetal heart beat seen at ultrasound examination at 6–8 weeks, expressed per started cycle. Secondary outcomes were the number of oocytes retrieved and number of metaphase II (MII) oocytes, implantation rate (defined as the number of gestational sacs observed divided by the number of embryos transferred), and live birth rate (defined as the number of deliveries per started cycles).

### Protocol

This study was exempt from institutional review board approval because it did not involve any human intervention. We adhered to the Preferred Reporting Items for Systematic Reviews and Meta-Analyses (PRISMA) guidelines [17].

### Study selection

First, the titles and abstracts of all articles were screened. Duplications were removed using both Endnote online software and manually. Disagreements were resolved by discussion among authors. The full texts of eligible articles published were subsequently obtained. The grey literature, namely, unpublished studies and those published outside widely available journals, case reports, conference proceedings, doctoral theses, dissertations, etc., was not considered [18] (Additional file 1: Table S1).

### Bias assessment

Three authors (IS, PD and AF) independently assessed the risk of bias of the studies eligible for the review using the checklist created by the Cochrane Menstrual Disorders and Subfertility Group [19]. The quality of allocation concealment was graded as adequate (A), unclear (B) or inadequate (C). Non-randomized trials were assessed using the Newcastle–Ottawa Scale (NOS) and each study was judged on three issues: selection of the study group, comparability between groups, and ascertainment of exposed and non-exposed cohorts [20].

Risk of bias across the studies was assessed by multiple analyses (Additional file 2: Table S2). Funnel plots of the primary outcome were evaluated both visually and formally with the ‘trim and fill’ method and the Egger test [21, 22].

### Quantitative analysis

Statistical analysis was carried out using Review Manager 5.3 (The Nordic Cochrane Centre, The Cochrane Collaboration). Categorical data were combined with a pooled odds ratio (OR) using the Mantel-Haenszel method. Continuous data were combined with weight mean differences (WMD) using the inverse variance method. The meta-analysis was conducted using the fixed-effect-model (FEM) or the random effect model (REM). REM was used in case of significant heterogeneity among studies. Heterogeneity was assessed using the percentage of total variation in the estimated effect across studies (I^2^). An I^2^ value > 50% indicates substantial heterogeneity. *P* values < 0.05 were considered statistically significant.

### Subgroup and sensitivity analysis

Subgroup analyses were carried out by study type, namely, randomized controlled trials and non-randomized controlled trials to identify potential sources of heterogeneity. Sensitivity analysis was carried out to assess the leverage of studies judged to be at high risk of bias (Additional file 3: Table S3). In detail, the risk of bias was evaluated considering the following issues: study design; imprecision (effect size with wide confidence interval), concerns regarding random sequence generation and allocation concealment.

## Results

### Study selection and characteristics

**Fig. 1:**
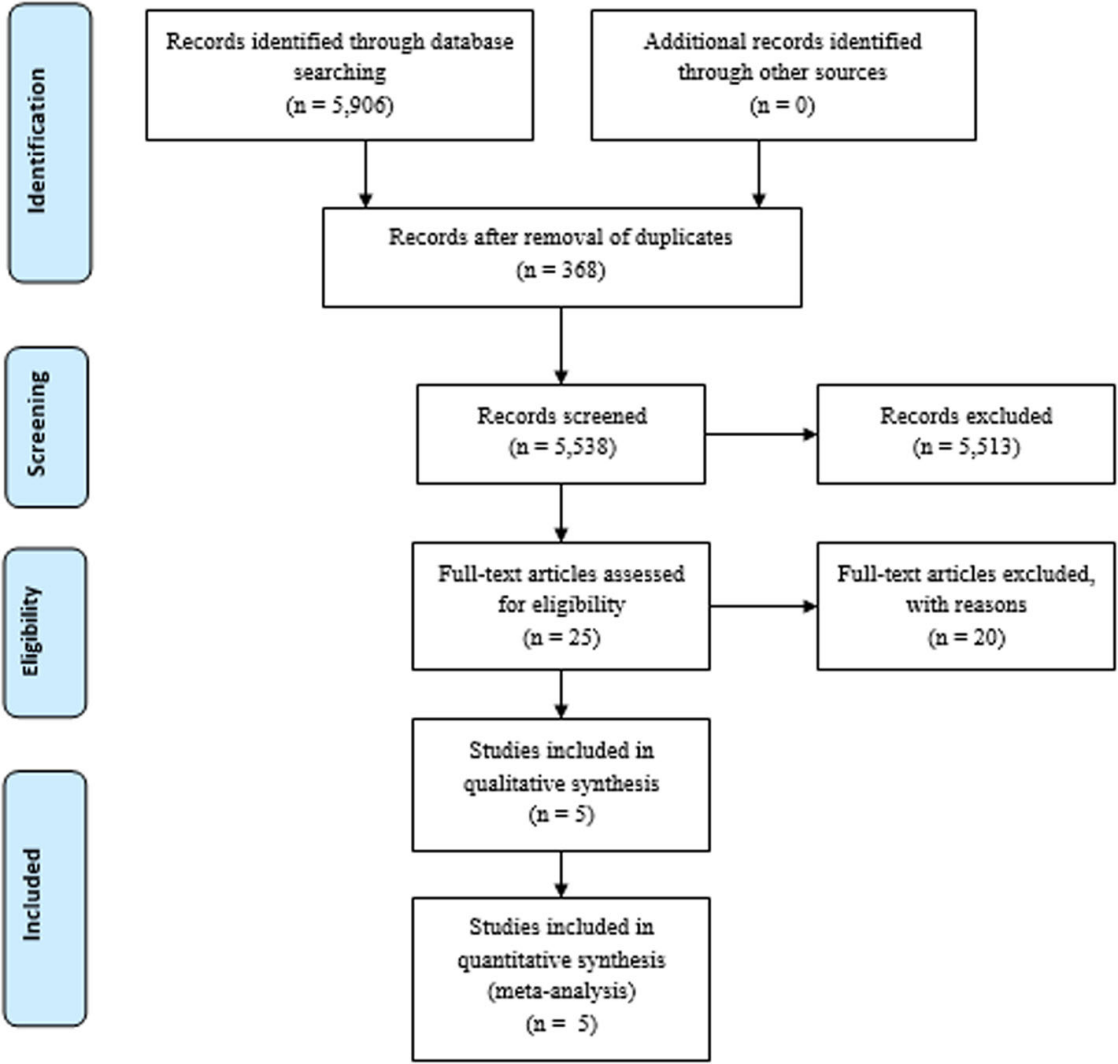
Study flow chart according to PRISMA guidelines

A total of 5906 items were identified in the Medline/PubMed (*N* = 3670) and Scopus databases (*n* = 2236) (Fig. 1). Duplications were removed by Endnote Online (*N* = 368). Abstracts and titles (*N* = 5538) were reviewed by two authors (AC, FC). Disagreements were resolved by discussion with senior authors (CA, SE, GD). Twenty-five full-text papers were assessed for eligibility. Twenty studies were excluded because they did not fulfill the inclusion criteria [23–42] (Additional file 4: Table S4). Five studies met the inclusion criteria and were included in our review. The characteristics of the studies and the risk of bias are reported in Table 1 and Additional file 2: Table S2.

**Table 1:** Characteristics of studies included

Reference	Study type	Period of observation	Country	Population	Intervention	Comparison	Effects of rLH supplementation
Lisi et al. 2002	RCT subgroup analysis	n.a.	Italy (single-centre)	*N* = 453 Included: Age not specified BMI ≥ 18 and ≤ 35 kg/m2 Regular menses Hyporesponse: defined as higher rFSH dosage consumption (> 2500 IU; *n* = 8) Excluded: Abnormal karyotype Any known endocrinopathy	rFSH + rLH rLH started at stimulation day 7 rLH dosage 75 IU	rFSH	Higher implantation rate
Ferraretti et al. 2004	RCT	January 2002 to April 2003	Italy (single-centre)	*N* = 126 Included: Age ≤ 37 years BMI ≤ 27 kg/m2 Normal uterine cavity Normal karyotypes Hypo-response defined as a “plateau on follicular growth” at stimulation days 7–10 in patients with > 10 antral follicles	*N* = 54 rFSH + rLH rLH started from mid-follicular phase rLH dosage 75 IU–150 IU	*N* = 50 rFSH step-up	Higher number of oocytes retrieved, implantation rate and pregnancy rate per embryo transfer
De Placido et al. 2005	RCT	February to December 2003	Italy (multi-centre)	*N* = 229 Included Age 18–37 years Basal FSH ≤ 9 IU/L BMI 18–28 kg/m^2^ Hypo-response defined as (i) E2 < 180 pg/ml and (ii) At least 6 follicles between 6 and 10 mm, but no follicles > 10 mm at stimulation day 8 Excluded: PCOS Endometriosis stage III-IV (AFS criteria) Endocrine disorders Autoimmune disorders > 2 previously unsuccessful IVF-ICSI Single ovary	*N* = 59 rFSH + rLH rLH started at stimulation day 8 rLH dosage 150 IU	*N* = 58 rFSH step-up	Higher number of oocytes retrived and MII oocytes
Ruvolo et al. 2007	RCT	September 2004 to February 2005	Italy (single-centre)	*N* = 42 Included Average age: 33.00 years (Group A) 36.33 years (Group B) BMI < 28 kg/m2 Basal FSH (< 12 IU/mL) Hypo-response defined as (i) E2 < 180 pg/ml and (ii) At least 6 follicles between 6 and 10 mm, but no follicles > 12 mm at stimulation day 8 and previous stimulation with > 3000 IU of rFSH	*N* = 24 rFSH + rLH rLH started at stimulation day 8 rLH dosage 75 IU–150 IU	*N* = 18 rFSH	Higher pregnancy rates and implantation rate
Yilmaz et al. 2015	Prospective study	January 2009 to April 2011	Turkey (single centre)	*N* = 137 Included: Age 23–39 BMI 18–30 kg/m^2^ FSH ≤ 12UI): Hypo-response defined as (i) E2 < 180 pg/ml and (ii) At least 6 follicles between 6 and 10 and no follicles > 10 mm at stimulation day 7 Excluded: Endometriosis Stage III-IV Single ovary PCOS Ovarian cyst	N = 50 rFSH + rLH rLH started at stimulation day 7 rLH dosage 75 IU	*N* = 35 rFSH step-up	Higher pregnancy and implantation rate

*n.a*. not available, *rFSH* recombinant FSH *rLH*: recombinant LH, ≈ comparable, *BMI* body mass index, *PCOS* polycystic ovarian syndrome

### Summary of results

#### Clinical pregnancy rate

**Fig. 2:**
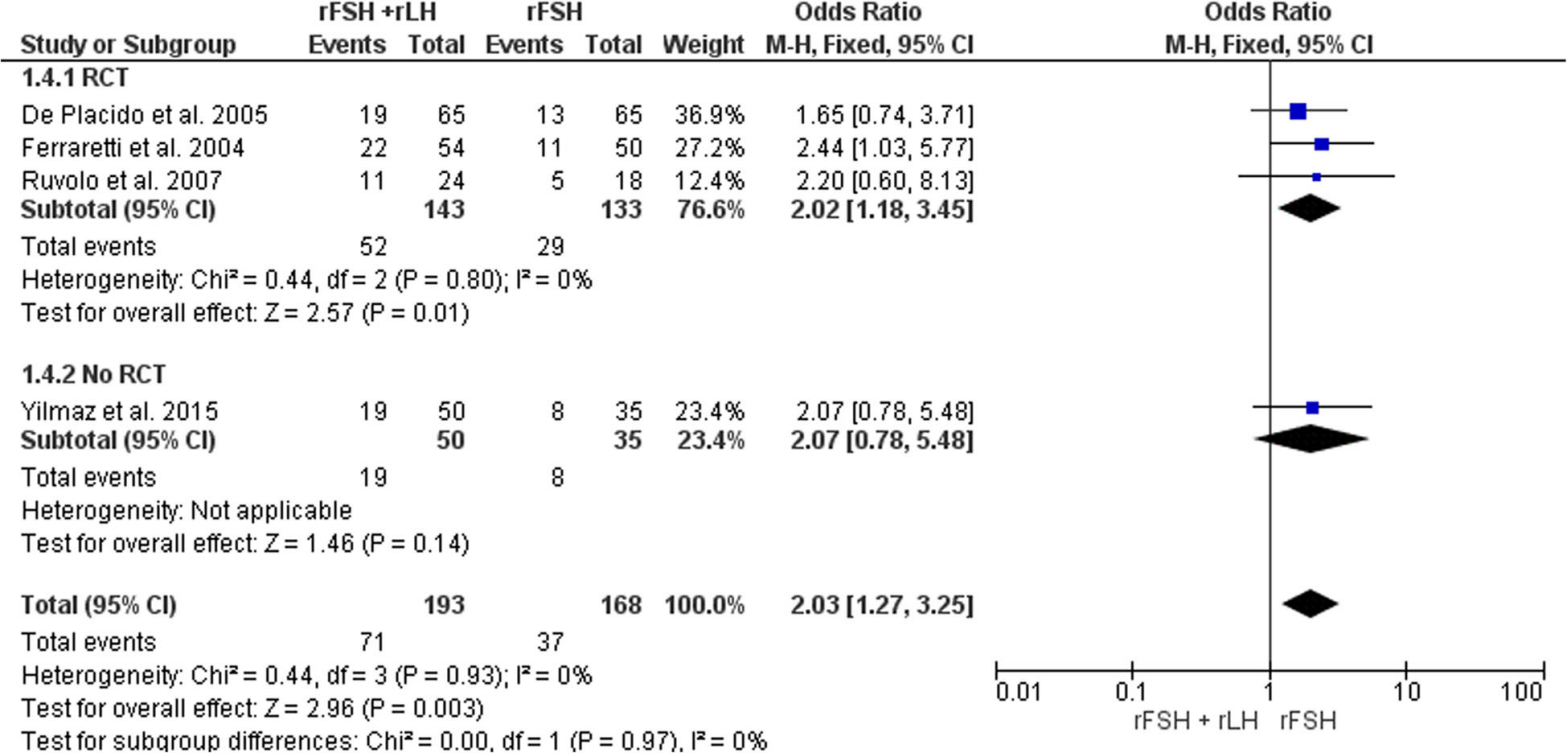
Forest plot of odds ratio for the clinical pregnancy rate in rFSH + rLH versus rFSH alone treatment

Clinical pregnancy rates were investigated in three RCTs [7–9] and in one cohort study [43] for a total of 361 participants (Fig. 2). The clinical pregnancy rate was significantly higher in the rFSH plus rLH group than in the rFSH alone group (OR 2.03, 95% CI 1.27–3.25, *P* = 0.003). The consistency in the direction of the effect, and the overlap in confidence intervals across studies increases confidence in the results. Sensitivity analysis indicated that removing the papers that were considered to have a high risk of bias [9, 43] did not affect the pooled effect size.

#### Number of oocytes retrieved

**Fig. 3:**
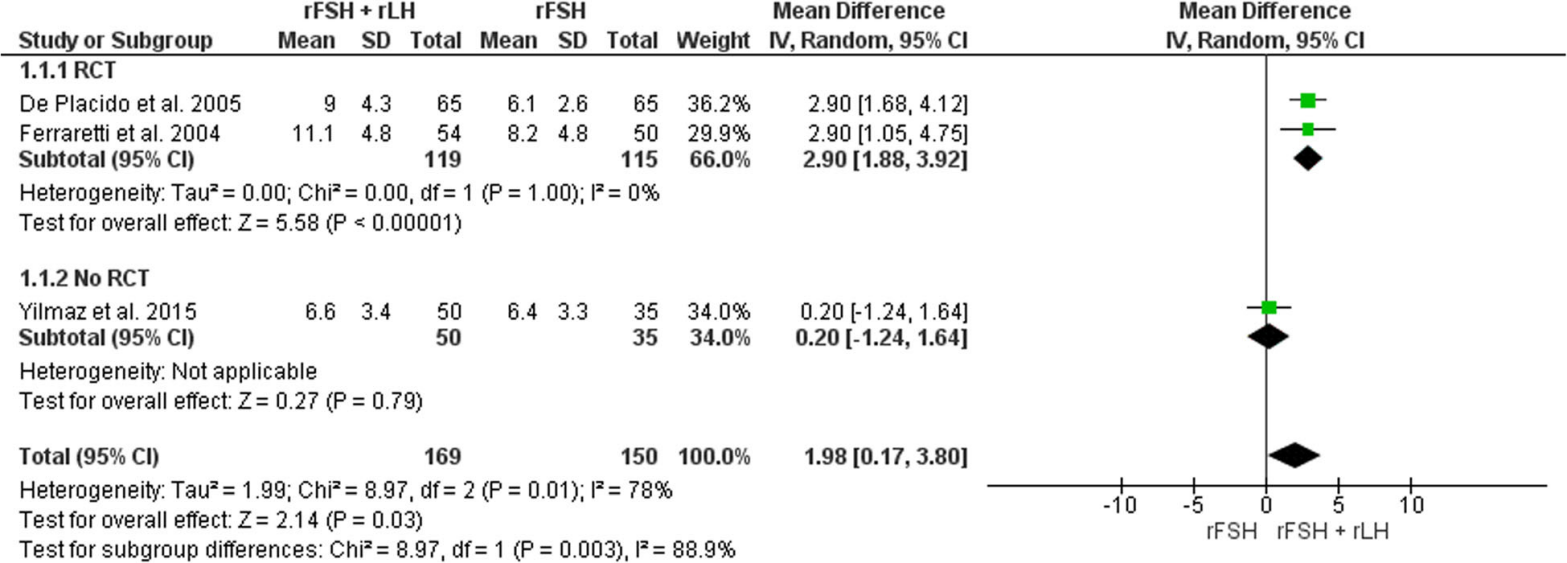
Forest plot of weight mean difference for the number of oocytes retrieved in rFSH + rLH versus rFSH alone treatment

Two RCTs [7, 8] and one cohort study [43], for a total of 319 participants, were analyzed (Fig. 3). The estimated pooled increase in the number of oocytes retrieved was 1.98 (WMD 1.98, 95% CI 0.17 to 3.80, I2 = 78%; *P* = 0.03), thus indicating a positive association between the use of rLH supplementation and number of oocyte retrieved in hypo-responders. To assess the cause of the heterogeneity, subgroup analysis was performed according to by study type. Heterogeneity was reduced to 0% in the RCT studies, suggesting that the difference between studies is explained by study type. The observed pooled effect size was larger for RCTs, with a higher number of oocytes retrieved compared to non-RCT (*P* = 003; Fig. 3). Sensitivity analysis indicated that removal of the study that had a high risk of bias [43] had no material effect on the results.

#### Number of metaphase II oocytes

Two RCTs [7, 9] and one cohort study [43], for a total of 257 subjects, were pooled. Additional file 5: Figure S1). Overall, no significant difference was observed between patients stimulated with rFSH + rLH and those with rFSH monotherapy in terms of number of MII oocytes retrieved (WMD 0.61, 95% CI -2.08 to 3.31; I2 = 90%). In subgroup analysis, the study type did not explain the heterogeneity between studies. Sensitivity analysis indicated that removal of the study with a high risk of bias [43] did not affect the pooled effect estimates.

#### Implantation rates

**Fig. 4:**
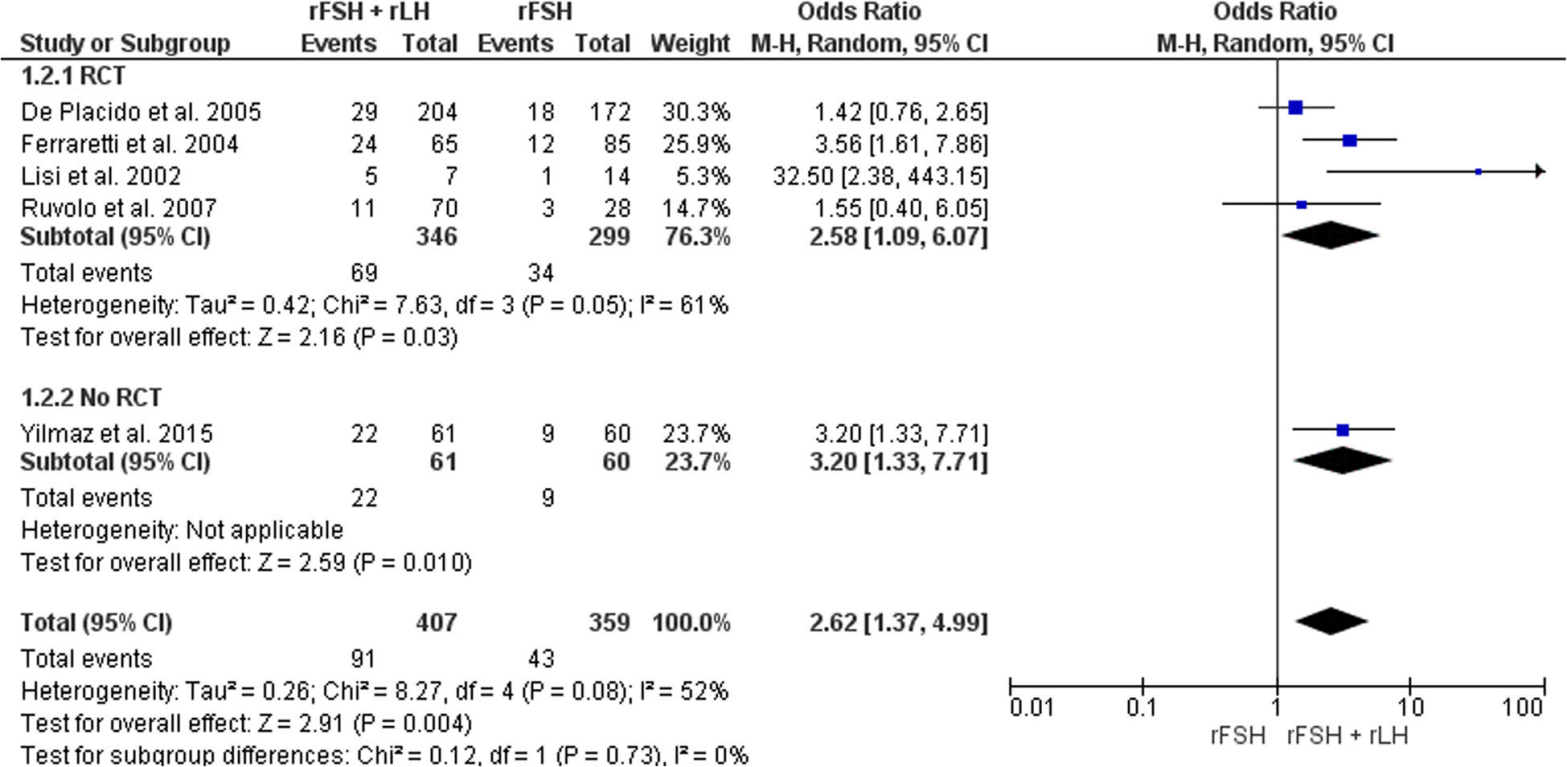
Forest plot of odds ratio for implantation rate in rFSH + rLH versus rFSH alone treatment

Four RCTs [7–9, 44] and one cohort study [43] for a total of 766 subjects, were analyzed (Fig. 4). The estimated pooled increase in implantation rates was 2.62 (95% CI OR 1.37–4.99, I2 = 52%, *P* = 0.004) favoring the rFSH + rLH group versus the rFSH alone group. The heterogeneity estimates were not materially affected by performing analyses separately by study type. However, heterogeneity in low risk of bias studies was reduced to 40% compared to 52% in the overall analysis (Additional file 3: Table S3), which suggests that some of the differences between studies are explained by study quality. These results are therefore consistent in suggesting a positive association between rLH supplementation and implantation rates, but they also indicate the need for additional studies to confirm the size of this association. Sensitivity analysis indicated that removal of studies with a high risk of bias [43, 44] did not affect the pooled effect estimates.

#### Live birth rates

Live birth rates were investigated in only one RCT [8]. As shown in Additional file 6: Figure S2, live birth rates were significantly higher in the rFSH + rLH group than in the rFSH alone group (OR 2.44, 95% CI 1.03–2.77, *P* = 0.04).

#### Miscarriage rate

Two RCTs [7, 8] and one cohort study [43] for a total of 319 participants, were pooled in this meta-analysis (Additional file 7: Figure S3). Overall, there was no significant difference in the estimated pooled miscarriage rate between patients stimulated with rFSH + rLH and patients on rFSH monotherapy (OR 1.01, 95% CI 0.52–1.99; I2 = 0%). Subgroup analysis demonstrated that study type had no material effect on the results. Similarly, sensitivity analyses showed that the removal of the study with a high risk of bias [43] did not affect the pooled effect size.

### Risk of bias across studies

The risk of a significant bias across studies regarding the primary outcome (clinical pregnancy rate) was excluded by Egger’s test (*P* = 0.642), which is confirmed by visual inspection of the funnel plots and the trim and fill method (Additional file 8: Figure S4).

## Discussion

### Summary of evidence

This meta-analysis indicates that women with ovarian resistance to exogenous gonadotropin who undergo COS for ART, the so-called “hypo-responders”, might benefit from rLH supplementation. The use of rLH supplementation was associated with increased clinical pregnancy, number of oocytes retrieved, and implantation rates, whereas no effect was noted concerning the number of MII oocytes and risk of miscarriage. The effect on live birth was less clear as to date, only one trial has reported LBRs in hypo-responders co-treated with rLH versus rFSH monotherapy during COS [8].

### Interpretation of results and clinical considerations

The concept of hypo-response to exogenous gonadotropin was introduced over a decade ago to describe women who have an impaired response to ovarian stimulation [5, 45, 46]. These normo-ovulatory and normo-gonadotropic women differ from classical poor responders in the sense that they are usually younger and have a normal age-matched ovarian reserve [13]. This ovarian resistance can clinically manifest as an “initial slow response” or “stagnation” in follicle growth during ovarian stimulation with FSH monotherapy [7, 8]. In particular, stagnation was defined by an absence or only marginal growth of both follicles and estradiol levels during OS. In other cases, a hypo-response can be retrospectively diagnosed in women who require higher-than-expected doses of gonadotropins on the basis of age, BMI and ovarian reserve [9, 47]. The trends seen in this meta-analysis are consistent with a beneficial effect of rLH supplementation during ovarian stimulation.

The mechanism by which rLH exerts its beneficial effect in hypo-responders is not fully understood. However, the fact that these patients and normal responders to gonadotropin stimulation share similar phenotypic characteristics suggests a genotype-based mechanism [10, 12, 48–50]. In other words, these women might have a peculiar genotype profile that could influence their response to ovarian stimulation. Since hypo-responders exhibit serum LH levels comparable to those of normal responders, it has been hypothesized that either the endogenous LH molecule or the LH receptor, or both, are functionally deficient and, therefore, implicated in the pathogenesis of hypo-response. Indeed, it has been reported that patients with a less functional common LH β chain variant have an increased resistance to gonadotropin stimulation [11, 51]. Furthermore, preliminary data show that even specific LH receptor polymorphisms could influence ovarian response during COS [52, 53]. In a previous study, we found that the prevalence of hypo-response was higher in carriers of the Serine variant of a common FSH-R polymorphism than in wild-type haplotypes [54]. Polymorphism of the FSH-R promoter was also associated with an impaired response to ovarian stimulation, i.e., higher consumption of exogenous gonadotropin in A allele carriers than in G allele carriers [55, 56].

It is, therefore, biologically plausible that LH supplementation in women with genetic polymorphisms involving gonadotropins and their receptors might overcome the genetically determined ovarian resistance to gonadotropin stimulation. In line with this hypothesis, Ramaraju et al. observed that both women heterozygous or homozygous for the G allele required higher doses of rLH supplementation during OS than those without these genetic traits; in their study, the use of rLH supplementation resulted in increased clinical pregnancy rates [57]. The added LH during stimulation acts on theca cells and increases androgen synthesis, which in turn improve FSH receptor sensitivity during COS [58–60]. This hypothesis should be verified in specific trials and could be a fruitful research topic.

As for the type of LH activity, rLH seems to be associated with better outcomes in hypo-responders than hMG. Indeed, a significantly lower implantation rate was observed in hypo-responders treated with hMG than with rLH [8]. This observation might be explained by the differential effect of LH activity provided by LH and hCG-containing drugs on the endometrium. Current evidence suggests that hCG (versus rLH) might adversely affect the endometrial function [61, 62]. Indeed, this phenomenon might explain why ongoing pregnancy rates are higher in frozen embryo transfer (FET) using natural cycles with spontaneous LH surge compared to natural cycles with exogenous hCG [63].

Additionally, there are differential biological activity of these two molecules on granulosa cells. Recombinant LH exerts both proliferative and antiapoptotic actions through phosphorylated extracellular-regulated kinase 1/2 (pERK1/2) and phosphorylated AKT signals [64]. By contrast, LH activity provided by hCG, contained in hMG formulations, displays a steroidogenic and proapoptotic effect mediated by cAMP and protein kinase A (PKA) [64]. These molecular differences might translate in improved pregnancy success among IVF patients treated with rLH rather than hMG, as shown in a 2017 large meta-analysis [14].

### Limitations and strengths

The main limitation of this study is the low number of trials conducted thus far. Ovarian resistance to gonadotropin stimulation remains a largely undervalued issue in the reproductive field. This may be because until recently, most clinicians did not consider the issue of hypo-responsiveness if the number of oocytes retrieved after stimulation was consistent with women’s reproduction potential and the antral follicle count at the beginning of stimulation. Indeed, previous systematic reviews investigated the role of rLH supplementation without distinguishing hypo-response from other conditions such a poor or normal response [19, 65]. In a 2014 meta-analysis for a total of 1129 ART cycles in POR patients supplemented or not with rLH illustrates this phenomenon [66]. In fact, it was noted that more oocytes were retrieved in rLH-supplemented cycles than with rFSH monotherapy (12 studies, *n* = 1077; weighted mean difference + 0.75 oocytes; 95% CI 0.14–1.36). In that study, the use of rLH supplementation also improved clinical pregnancy rates (14 studies, *n* = 1179; relative risk [RR] 1.30; 95% confidence interval [CI] 1.01–1.67; intention-to-treat population [ITT] population). Nevertheless, a careful examination of the included studies reveals that the beneficial effect of rLH was more pronounced in studies involving hypo-responders than in those with classic POR. The inclusion of studies involving hypo-responders in the Lehert et al. (2014) review thus explains the overall favorable results observed with rLH supplementation in the POR patient.

The merit of our review is that, albeit at the cost of a low number of observations, it focused on prospective clinical trials involving the specific subgroup of hypo-responders. Moreover, the low heterogeneity among trials utilized in our meta-analyzes concerning the primary outcome provides evidence that the observed effect was causal. Nevertheless, heterogeneity was high among most secondary outcomes, which might be partially explained by study type and quality, as the subgroup and sensitivity analyses demonstrated. Furthermore, different protocols and rLH dosages were used in the studies examined. It was not possible to evaluate outcomes according to the dosage and time of LH administration due to the low number of studies. Only one trial demonstrated that 150 IU of rLH result in more oocytes retrieved and a higher percentage of mature oocytes than do 75 IU in a long GnRH agonist protocol [23]. Along the same lines, the effect of rLH supplementation in hypo-responders undergoing OS with GnRH antagonists remains to be established, as the trials included in this meta-analysis utilized pituitary suppression with GnRH agonist. Despite the low number of studies and high heterogeneity in some of the outcome measures, there was no evidence of publication bias. Furthermore, sensitivity analyses demonstrated minimal differences when studies with high risk of bias were excluded, thus indicating our results are conservative.

### Future research

Ovarian resistance to exogenous gonadotropin still represents an undervalued topic in the reproductive field. There is a lack of a standardized definition and limited knowledge about its pathophysiology mechanisms. Two years ago, hypo-responsiveness to gonadotropin stimulation was included in a novel stratification system, designated the POSEIDON criteria, for infertility patients undergoing ART [4, 67, 68]. Novel markers that accurately reflect the “dynamic” nature of follicular growth in response to exogenous gonadotropin are under investigation, an example being the follicle-to-oocytes index (FOI), which is the ratio between the number of oocytes retrieved and AFC at the beginning of stimulation [69]. A better understanding of the effect exerted by genetic polymorphisms of gonadotropins and their receptors, and subsequently COS outcomes, will help to improve treatment and counseling for patients seeking ART.

Additional studies, in particular those that assess various LH supplementation doses and regimes, including GnRH antagonists and cumulative LBR as the main outcome measure, are required. Such studies might increase the precision of the estimated effect sizes, thus allowing better appraisal of the clinical importance of our findings.

## Conclusion

In conclusion, our analyses indicate that women with a previous hypo-response to exogenous FSH stimulation benefit from LH supplementation as a means of increasing clinical pregnancy, implantation, and number of oocytes retrieved. LH supplementation might be added during COS on the same cycle to rescue an ongoing hypo-response or in a subsequent cycle. Further research is required to quantify the effect of LH supplementation more precisely and to evaluate the clinical utility of LH supplementation in hypo-responders using the GnRH antagonist protocol.

## Additional files

Additional file 1: **Table S1.** Search and selection strategy. (DOCX 11 kb)

Additional file 2: **Table S2.** Quality of study included. (DOCX 12 kb)

Additional file 3: **Table S3.** Sensitivity analysis. (DOCX 13 kb)

Additional file 4: **TableS4.** Excluded studies with reasons. (DOCX 12 kb)

Additional file 5: **Figure S1.** Forest plot of weight mean difference for the number of metaphase II oocytes in rFSH + rLH versus rFSH alone treatment. (TIF 243 kb)

Additional file 6: **Figure S2.** Forest plot of odds ratio for live birth rate in rFSH + rLH versus rFSH alone treatment. (TIF 107 kb)

Additional file 7: **Figure S3.** Forest plot of odds ratio for miscarriage in rFSH + rLH versus rFSH alone treatment. (TIF 263 kb)

Additional file 8: **Figure S4.** Funnel-plots and “trim and firm” analysis of primary outcome. (TIF 948 kb)
