# Correction to: The role of recombinant LH in women with hypo-response to controlled ovarian stimulation: a systematic review and meta-analysis

**DOI:** 10.1186/s12958-019-0475-x

**Published:** 2019-03-14

**Authors:** Alessandro Conforti, Sandro C. Esteves, Francesca Di Rella, Ida Strina, Pasquale De Rosa, Alessia Fiorenza, Fulvio Zullo, Giuseppe De Placido, Carlo Alviggi

**Affiliations:** 1 Department of Neuroscience, Reproductive Science and Odontostomatology, University of Naples Federico II, Naples, Italy; 2 ANDROFERT, Andrology and Human Reproduction Clinic, Campinas, Brazil; 3 Department of Senology, Medical Oncology, National Cancer Institute, IRCCS “Fondazione G.Pascale”, Naples, Italy; 4 Unit of Obstetrics and Gynaecology, Department of Experimental and Clinical Medicine, Magna Graecia University of Catanzaro, Catanzaro, Italy; 5 Istituto per l’Endocrinologia e l’ Oncologia Sperimentale (IEOS) Consiglio Nazionale delle Ricerche, Naples, Italy

**Correction to: Reproductive Biology and Endocrinology (2019) 17:18**

**https://doi.org/10.1186/s12958-019-0460-4**

Following publication of the original article [1], the authors would like to apologize for an error in Fig. 2 describing clinical pregnancy rate comparison between treatment arms. The authors would like to reassure the readers that this minor type error does not affect any other content and the main findings of the article. The correct figure is presented below.

**Fig. 2:**
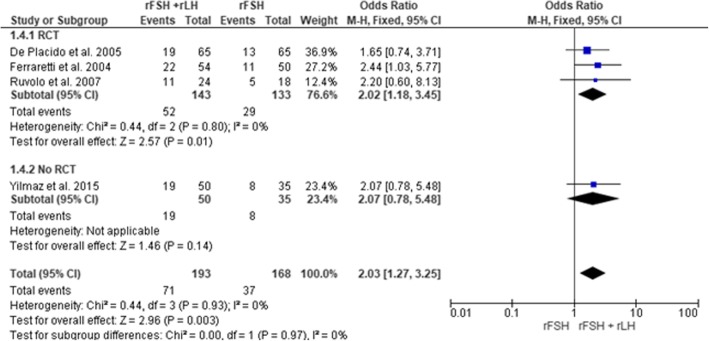
Forest plot of odds ratio for the clinical pregnancy rate in rFSH + rLH versus rFSH alone treatment
