# Supplementary material for: The role of recombinant LH in women with hypo-response to controlled ovarian stimulation: a systematic review and meta-analysis
Source: Reprod Biol Endocrinol. 2019 Feb 6;17:18. doi: 10.1186/s12958-019-0460-4 (PMC6366097; doi:10.1186/s12958-019-0460-4)
Supplement: Supplementary file 1 — Table S1. Search and selection strategy. (DOCX 11 kb) [file 12958_2019_460_MOESM1_ESM.docx]

**Table S1: Search and selection strategy**

| **Databases searched** | Pubmed/Medline, Scopus |
| --- | --- |
| **Other sources** | Additional studies were identified through references identified from included studies and reviews. Grey literature no included |
| **Search Key words** | Exposure: [recombinant LH OR rLH OR rhLH OR luteinizing hormone]  AND [Assisted reproductive technologies OR ART OR In vitro fertilization OR IVF OR steady response OR hypo-response] |
| **Inclusion criteria** | randomized controlled trials and prospective trials |
| **Exclusion criteria** | case reports, books, unpublished articles, retrospective trials, prospective trials, no English papers |
