# Supplementary material for: The role of recombinant LH in women with hypo-response to controlled ovarian stimulation: a systematic review and meta-analysis
Source: Reprod Biol Endocrinol. 2019 Feb 6;17:18. doi: 10.1186/s12958-019-0460-4 (PMC6366097; doi:10.1186/s12958-019-0460-4)
Supplement: Supplementary file 2 — Table S2. Quality of study included. (DOCX 12 kb) [file 12958_2019_460_MOESM2_ESM.docx]

**Table S2 a.** Quality of RCTs included

| **Study** | **Grade for concealment** | **Method of concealment** | **Randomization methods** | **ITT** | **Dropouts** | **Baseline comparability** |
| --- | --- | --- | --- | --- | --- | --- |
| De Placido et al.  2005 | A | Unknown | Comp.  generated random table  numbers | Yes | 13 added to denominator for analysis | Comparable |
| Ferraretti et al.  2004 | B | Unknown | Unknown | Yes | 4, added to denominator analysis | Comparable |
| Lisi et al.  2002 | C | Unknown | Unknown | No | 0 | Comparable |
| Ruvolo et al.  2007 | A | Unknown | Comp.  generated tables | No | 0 | Comparable |

**Table S2 b.** Quality of no RCTs studies included

| **Study** | **Selection** | **Comparability** | **Outcome** | **Total score** |
| --- | --- | --- | --- | --- |
| Yilmaz et al. 2016 | **** | * | *** | 8 |
