# Supplementary material for: The role of recombinant LH in women with hypo-response to controlled ovarian stimulation: a systematic review and meta-analysis
Source: Reprod Biol Endocrinol. 2019 Feb 6;17:18. doi: 10.1186/s12958-019-0460-4 (PMC6366097; doi:10.1186/s12958-019-0460-4)
Supplement: Supplementary file 3 — Table S3. Sensitivity analysis. (DOCX 13 kb) [file 12958_2019_460_MOESM3_ESM.docx]

| **Outcome** | **All studies** |  | | **Sensitivity analysis^1^** | | | **Results** |
| --- | --- | --- | --- | --- | --- | --- | --- |
|  | **N. Studies/**  **participants** | **Effect size [95%CI]** | **I^2^** | **N. Studies/**  **participants** | **Effect size [95%CI]** | **I^2^** |  |
| Clinical pregnancy rate | 4/361 | 2.03[1.27-3.25] | 0% | 2/276^a^ | 2.02[1.18;3.45] | 0% | Not affected |
| N. oocytes retrieved | 3/319 | 1.98[0.17-3.80] | 78% | 2/234^b^ | 2.90[1.88;3.92] | 0% | Not affected |
| N. Metaphase II oocytes | 3/257 | 0.61[-2.08-3.31] | 90% | 2/272^b^ | 0.83[-3.83;5.50] | 92% | Not affected |
| Implantation rate | 5/766 | 2.62[1.37-4.99] | 52% | 3/624^c^ | 2.02 [1.06-3.82] | 40% | Not affected |
| Live birth rate | 1/104 | 2.44[1.03-5.77] | / | 1/104 | 2.44[1.03-5.77] | / | / |
| Miscarriage rate | 3/319 | 1.01[0.52;1.99] | 0% | 2/234^b^ | 0.82[0.36;1.31] | 0% | Not affected |
| ^1^Sensitivity analysis excluding studies judged to be at high risk of bias  ^a^ Yilmaz et al. for study design, Ruvolo et al. for imprecision  ^b^ Yilmaz et al. for study design  ^c^ Yilmaz et al. for study desing and Lisi et al. 2002 for imprecision and allocation concealment | | | | | | | |
