# Supplementary material for: The role of recombinant LH in women with hypo-response to controlled ovarian stimulation: a systematic review and meta-analysis
Source: Reprod Biol Endocrinol. 2019 Feb 6;17:18. doi: 10.1186/s12958-019-0460-4 (PMC6366097; doi:10.1186/s12958-019-0460-4)
Supplement: Supplementary file 4 — TableS4. Excluded studies with reasons. (DOCX 12 kb) [file 12958_2019_460_MOESM4_ESM.docx]

| ***Study*** | ***Reason for exclusion*** |
| --- | --- |
| De Placido et al. 2004 | Lack of a control group without LH supplementation |
| Westergard et al. 2000; De Placido et al. 2001; | Lack of a group using rLH supplementation during COS |
| Sills et al. 1999; Balasch et al. 2001; Cedrin-Durnerin I et al. 2004; Humaidan et al. 2004; Marrs et al. 2004; Sauer et al. 2004; Griesinger et al. 2005; Tarlatizis et al. 2006; Levi Setti et al. 2006; Garcia-Velasco et al. 2007; Nyboe Andersen et al. 2008; Bosch et al. 2011; | Enrolled patients did not fit into the criteria of hypo-responders |
| Musters et al. 2012; Humadain et al. 2017 | Enrolled patients comprised poor responders rather than hypo-responders |
| Lisi et al. 2005; Pezzuto et al. 2010; Lahoud et al. 2017; | Patients with LH suppressed during COS did not fit into the criteria of hypo-responders |

**Table S4**. Excluded studies with reasons
