# Supplementary figures and images for: The role of recombinant LH in women with hypo-response to controlled ovarian stimulation: a systematic review and meta-analysis
Source: Reprod Biol Endocrinol. 2019 Feb 6;17:18. doi: 10.1186/s12958-019-0460-4 (PMC6366097; doi:10.1186/s12958-019-0460-4)

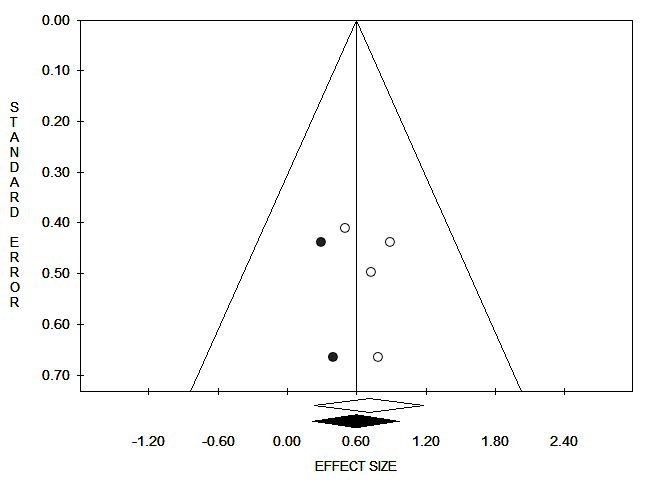

Supplement: Supplementary file 8 — Figure S4. Funnel-plots and “trim and firm” analysis of primary outcome. (TIF 948 kb) [file 12958_2019_460_MOESM8_ESM.tif]
